# Burkholderia Cepacia-Induced Bilateral Microbial Keratitis in a Wearer of Colored Cosmetic Contact Lenses

**DOI:** 10.7759/cureus.22840

**Published:** 2022-03-04

**Authors:** Maria Miura, Hiroshi Toshida

**Affiliations:** 1 Ophthalmology, Juntendo University School of Medicine, Tokyo, JPN; 2 Ophthalmology, Juntendo University Shizuoka Hospital, Shizuoka, JPN

**Keywords:** corneal ulcer, complication, colored cosmetic contact lens, keratitis, burkholderia cepacia

## Abstract

In this report, we present a case of ​​​​​​*Burkholderia cepacia* (*B. cepacia*)-induced bilateral microbial keratitis in a patient who wore colored contact lenses (CLs) on both eyes. The patient was a 19-year-old woman who presented to our hospital with pain, discharge, and photophobia affecting both eyes while she was wearing colored cosmetic CLs. There were corneal ulcers in both of her eyes without refractive correction. Although impairment of vision was not detected at the first visit, both eyes showed neovascularization of the upper part of the cornea and had relatively well-demarcated corneal ulcers associated with corneal stromal opacity. Cultures of cornea grew *B. cepacia* in both eyes. She received antimicrobial agents to which this organism was sensitive, resulting in the healing of the corneal ulcers with scarring after approximately one month. Improper fitting of colored cosmetic CLs, contamination of lenses or solutions, and inadequate lens care can be risk factors for developing this condition.

## Introduction

*Burkholderia cepacia* (*B. cepacia*) is an aerobic, oxidase-positive, Gram-negative bacillus found in water tanks and high-humidity wet environments [1]. In 1949, Burkholder initially identified it as a cause of onion rot. In humans, this microorganism is known as an opportunistic pathogen that causes pulmonary infection associated with cystic fibrosis [2]. Recently, the reports related to ocular infections have been gradually on the rise, especially regarding endophthalmitis after ocular surgeries [3-6], including intravitreous injection of anti-vascular endothelial cell growth factor agent [7]. *B. cepacia* has been reported to contaminate topical anesthetics and has also been detected in a cornea tissue bank for keratoplasty [8], as well as rigid gas permeable trial lenses and lens cases [9]. There are only a few reports in the literature regarding *B. cepacia* microbial keratitis related to contact lens (CL) wearing [10,11]. In this report, we discuss a case of *B. cepacia-induced* bilateral microbial keratitis in a wearer of colored cosmetic CLs.

## Case presentation

**Figure 1:**
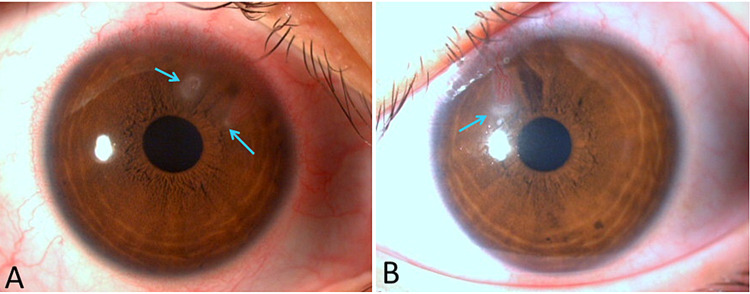
Microscopic image of the right eye (A) and the left eye (B) at the first visit There was neovascularization in the upper part of the cornea along with a relatively well-demarcated corneal ulcer in both eyes (arrows)

A 19-year-old woman presented to the Juntendo University Shizuoka Hospital with pain, discharge, and photophobia in both eyes. She wore colored cosmetic CLs. At the first visit, the visual acuity was 20/20 and uncorrectable in both eyes. Examination of the anterior segment revealed neovascularization in the upper part of the cornea along with a relatively well-demarcated corneal ulcer in the right eye (Figure 1). The assessment of the ocular media, ocular fundus, and intraocular pressure was unremarkable. Her personal and family medical history was not contributory. No microorganisms were detected on microscopy of corneal scrapings at the first visit. Therefore, corneal scrapings from both eyes and the CL-soaking solution in the lens cases for both eyes were subjected to bacterial culture. The patient had purchased her CLs at a variety store without undergoing an examination or obtaining a prescription from a clinic. The colored cosmetic CLs (Fairy Princess, Fairy Series, Vizionfocus Inc., Taiwan) were soft CLs (SCLs) for monthly exchange and made of hydroxyethyl methacrylate (HEMA). She used these lenses approximately seven hours daily for five to six days a week. The name of the multi-purpose solution (MPS) she used for lens care was unknown, and the lenses were not regularly cleaned by scrubbing. The MPS in the lens cases was not routinely changed adequately either.

After presenting to us, she discontinued wearing the lenses. The fitting pattern of CLs was tight in both eyes. Before the results of bacterial culture became available, empirical therapy was started by topical application of gatifloxacin, tobramycin, cefmenoxime, and pimaricin in both eyes four times daily. After one week of treatment, *B. cepacia* was detected in the cornea-scraped sample from the left eye and lens-soaking solution of the lens case for both eyes. Table 1 shows the results of the drug sensitivity testing.

**Table 1:** Antibacterial spectrum for detected Burkholderia cepacia MIC: minimum inhibitory concentration; S: sensitive; I: intermittent; R: resistant

Antibiotics	Abbreviation	MIC	Evaluation of antibacterial susceptibility
Ampicillin	ABPC	32	R
Piperacillin	PIPC	8	I
Ampicillin/sulbactam	ABPC/SBT	32	R
Cefazolin	CEZ	32	R
Cefotiam	CTM	32	R
Cefmetazole	CMZ	64	R
Ceftazidime	CAZ	4	S
Amikacin	AMK	64	R
Gentamicin	GM	16	R
Minocycline	MINO	1	S
Levofloxacin	LVFX	4	I
Gatifloxacin	GFLX	4	R
Sulfamethoxazole-trimethoprim	ST	19	S

**Figure 2:**
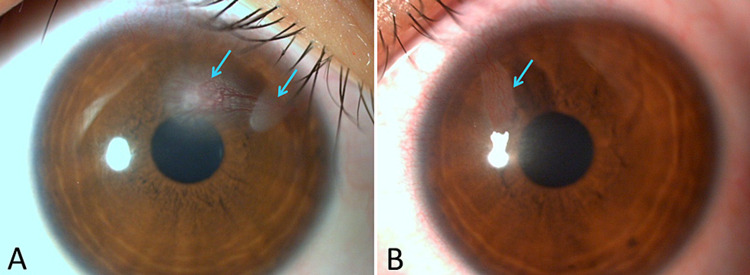
Microscopic image of the right eye (A) and the left eye (B) after one month of treatment Subepithelial opacity (arrows) and corneal neovascularization persisted. Hyperemia decreased compared to that at the first visit

The treatment was changed to topical application of levofloxacin four times daily because the microorganism showed sensitivity to this antimicrobial agent. And the other antimicrobial eye drops were discontinued because of drug resistance. After one month of treatment, corneal staining with fluorescein was found to be negative, and hence the infection was considered to have been cured. However, corneal opacity in the subepithelial layer and corneal neovascularization persisted (Figure 2), and topical application of 0.1% fluorometholone was added to suppress inflammation. After that, the patient did not consult our hospital again.

**Figure 3:**
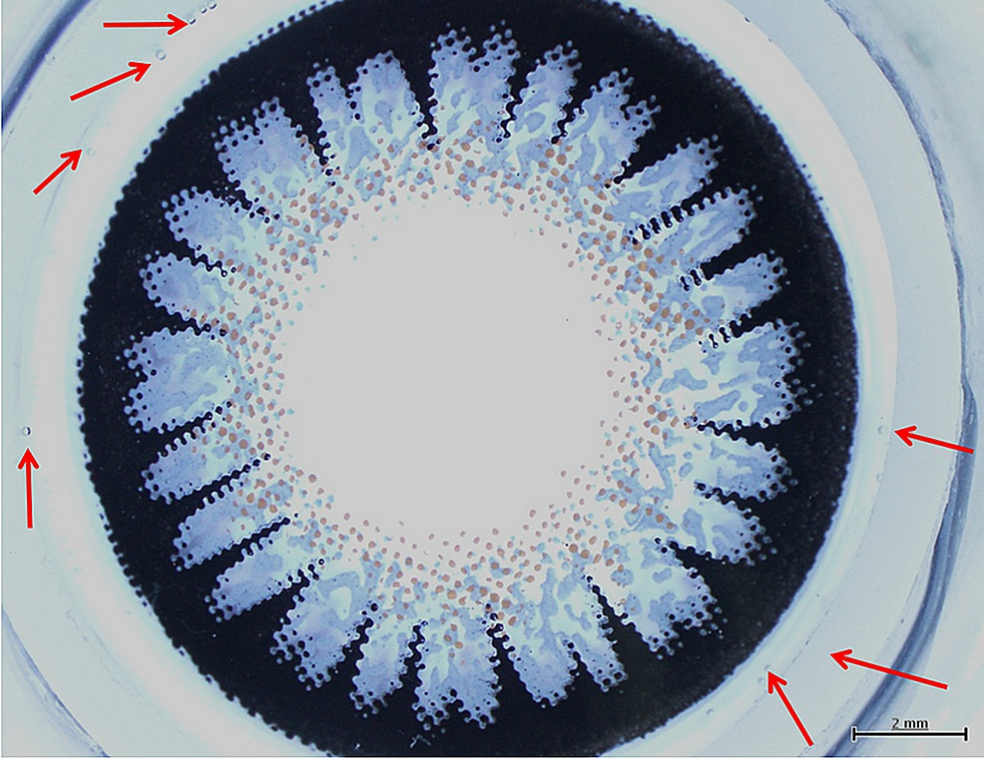
Color cosmetic contact lens of the patient for the left eye. The arrows show deposits on the contact lens

**Figure 4:**
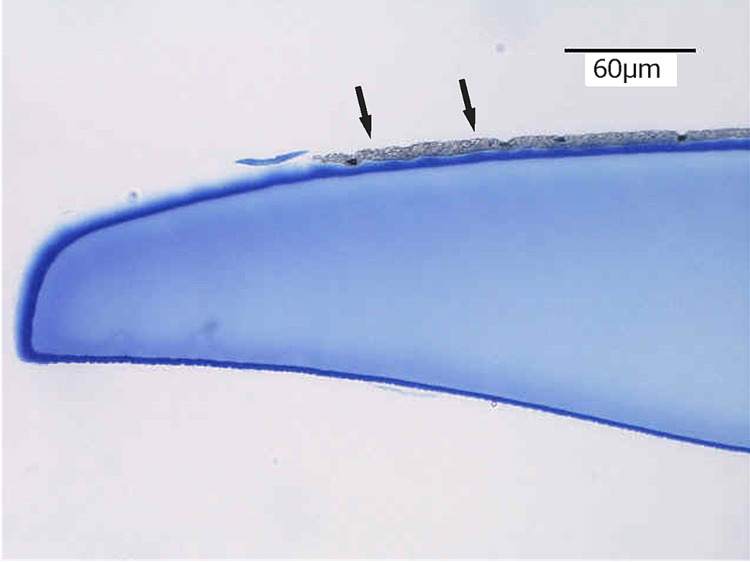
Color dye on the colored cosmetic contact lens in the front (arrows). The surface of the colored dye was not smooth

Her colored cosmetic CL was photographed (Figure 3) and observed by light microscopy stained by toluidine blue (Figure 4). The color dye pigments were on the lens surface in the front, and they did not seem smooth on the pigmented area.

## Discussion

Colored cosmetic SCLs have been associated with eye complications in many countries around the world [12]. These lenses can be purchased without a prescription in some countries and can be used without ophthalmological examination and lens-fitting by an ophthalmologist or optometrist in some countries. Therefore, there is the possibility that these CL fittings are not always proper. The main material that colored SCLs comprise is usually HEMA, which has low water content. So, colored SCLs tend to be insufficiently flexible. Mechanical stimulations to the cornea due to improper lens fitting can lead to damage to the corneal epithelial barrier, resulting in the occurrence of keratitis.

Generally, young healthy women, like our patient, are less likely to get infected as compared to persons with underlying diseases. However, cornea infection of both eyes developed in our patient. It is speculated that the lens-fitting was inappropriate in her case, and that is how she became susceptible to the risk of the cornea infection onset. She owned several samples of colored cosmetics CLs and did not receive advice about wearing them from a clinic. She also did not receive any instructions about lens care. Hence, her method of lens care was not appropriate, and she did not wash her lenses and lens case. Further, she never changed the MPS in the lens case. As for the MPS, the effect fades over time. In any case, such careless wearing and maintenance of the lenses turned out to be a risk factor, and the mechanical irritation to corneas due to poor fitting disrupted an epithelial barrier function and was thought to have caused *B. cepacia *infection.

*B. cepacia *prefers a wet environment, such as contaminated mouthwashes [13]. Unlike most bacteria, it shows high viability and develops resistance to solutions for sterilization and disinfection [14]. Because the inside of a CL case is always wet when storing the lenses, this microorganism could grow in the case even if a disinfecting lens care agent is being used. However, only a few case reports have been published related to CL-related keratitis due to *B. cepacia* [10,11]. One reason for this may be that* B. cepacia* is generally considered to be a pathogen associated with opportunistic pulmonary infection that does not cause problems in healthy people [15]. Another reason could be the difficulty in detecting *B. cepacia* by culture due to its slow growth. Once *B. cepacia* infection occurs, multiple drug resistance often develops in patients with pulmonary infection, resulting in necrotizing pneumonia in many cases [2]. In the ophthalmology field, previous reports about *B. cepacia* have suggested that eye infection is triggered by the disruption of the ocular surface barrier by surgeries including laser in situ keratomileusis [16], trauma, or CL complications. Once *B. cepacia* eye infection develops, it becomes relatively serious according to these earlier reports.

CL wearing is reportedly the main risk factor for infectious keratitis in Japan [17]. Recently, it has been observed that the major cause of serious CL-associated ocular complications is microbial keratitis, and Gram-negative bacilli and Acanthamoeba are the major pathogens in Japan [18]. In general, Gram-negative bacilli usually produce toxins that aggravate inflammation. In our patient, however, keratitis resolved rapidly after the patient’s antimicrobial therapy was changed based on the results of sensitivity testing. Our case emphasizes that it is essential to follow the fundamental rules of infection management, i.e., bacterial culture should be performed if an infection is suspected, and antimicrobial agents should be selected based on the results of sensitivity tests.

The oxygen permeability (Dk) required for wearing CLs all day long is 24.1. To achieve this with SCL, the mean lens thickness should be less than 33 micrometers; however, colored CLs are usually thicker than this, and oxygen permeability through the colored part is usually poor [19]. Hence, there is a possibility that inflammation in our case was aggravated by mechanical stimulation and infection coupled with hypoxic damage. In fact, our patient had corneal neovascularization in both eyes.

## Conclusions

We reported a rare case of infectious keratitis caused by *B. cepacia* infection in both eyes, which was associated with colored cosmetic CLs. The patient's keratitis was presumably induced by mechanical stimulation due to the tight-fitting of the CLs. *B. cepacia* prefers a wet environment and shows high viability and develops resistance to solutions for sterilization and disinfection, probably including MPS. Therefore, when opting for colored cosmetic CLs, a prescription from a doctor and choosing correct CLs based on the results of a proper eye examination and proper lens-fitting are important factors to be considered. Also, proper care of lenses and the lens case is also important to prevent complications from CL wearing.
